# Supplementary material for: Unravelling microalgal-bacterial interactions in aquatic ecosystems through 16S rRNA gene-based co-occurrence networks
Source: Sci Rep. 2023 Feb 16;13:2743. doi: 10.1038/s41598-023-27816-9 (PMC9935533; doi:10.1038/s41598-023-27816-9)
Supplement: Supplementary file 1 — Supplementary Information 1. [file 41598_2023_27816_MOESM1_ESM.docx]

# **Supplementary Data**

**Figure S1:** **Map indicating the sample collection sites for each EMP project.** Sample collection locations for each EMP project are shown on the world map. Geographic coordinates were drawn in R studio (v1.4.1106) using packages rworldmap v1.3-6 (https://rdrr.io/cran/rworldmap/).

**Figure S2:** **Major bacterial Phyla associated with microalgae in freshwater and marine environments**

Bars represent the number of bacterial nodes affiliated with each phylum. The total number of bacterial nodes identified to be co-occurring with microalgae in marine environments were 76 and freshwater environments were 311.

**Figure S3:** ***Planctomycetes* class *OM190* interacting with microalgal taxa in a marine (A) and freshwater (B) environment.**

Networks (A, B) created from a microalgal-bacterial module by selecting the first neighbours of the *OM190* to showcase the complex interactions with the neighbouring microalgal taxa. The edge width and node size are continuously mapped to edge weight and node degree. Marine and Freshwater environments represent EMP project ERP020022 and ERP016492, respectively. Nodes labelled at order level (If unclassified at the order level, higher taxonomic affiliations are provided). Network image was generated using Cytoscape v3.8 (https://cytoscape.org/).

**Table S1: Information on selected EMP studies.** Details on Qiita study title, study ids and ENA project accession number.

**Table S2: Correlations recovered in marine environments between microalgae and bacteria.** Summary of significant co-occurrences recovered from the marine environments. Full taxonomic affiliations of the microalgal and bacterial nodes are provided.

**Table S3: Correlations recovered in freshwater environments between microalgae and bacteria.** Summary of significant co-occurrences recovered from the freshwater environments. Full taxonomic affiliations of the microalgal and bacterial nodes are provided.
