# Supplementary figures and images for: Unravelling microalgal-bacterial interactions in aquatic ecosystems through 16S rRNA gene-based co-occurrence networks
Source: Sci Rep. 2023 Feb 16;13:2743. doi: 10.1038/s41598-023-27816-9 (PMC9935533; doi:10.1038/s41598-023-27816-9)

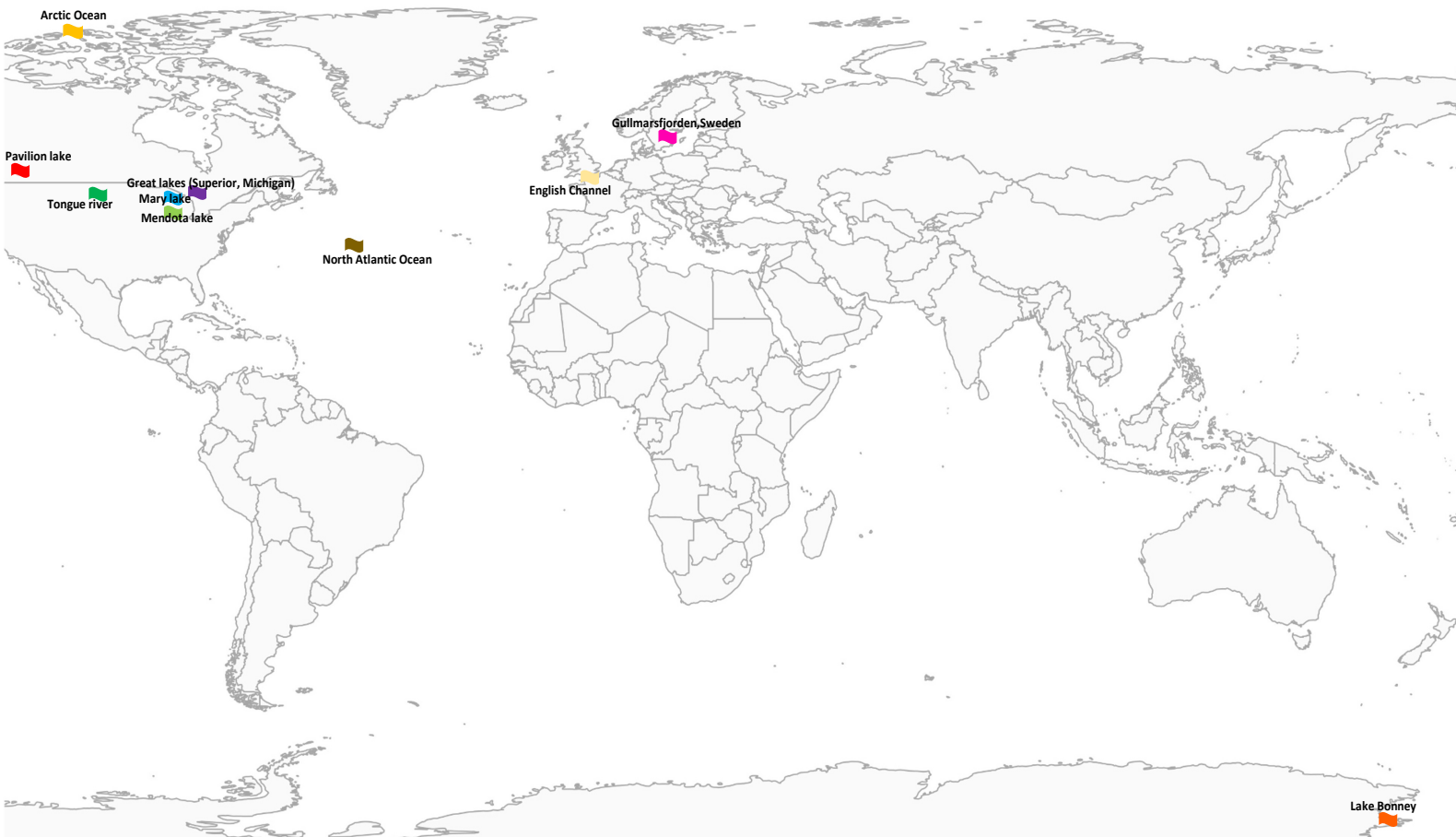

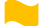 ERP020022

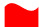 ERP020021

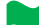 ERP016468

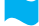 ERP016854

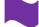 ERP016492

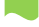 ERP016591

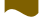 ERP016287

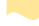 ERP016541

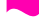 ERP021691

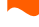 ERP020508

Supplement: Supplementary file 2 — Supplementary Information 2. [file 41598_2023_27816_MOESM2_ESM.pdf]

### Freshwater

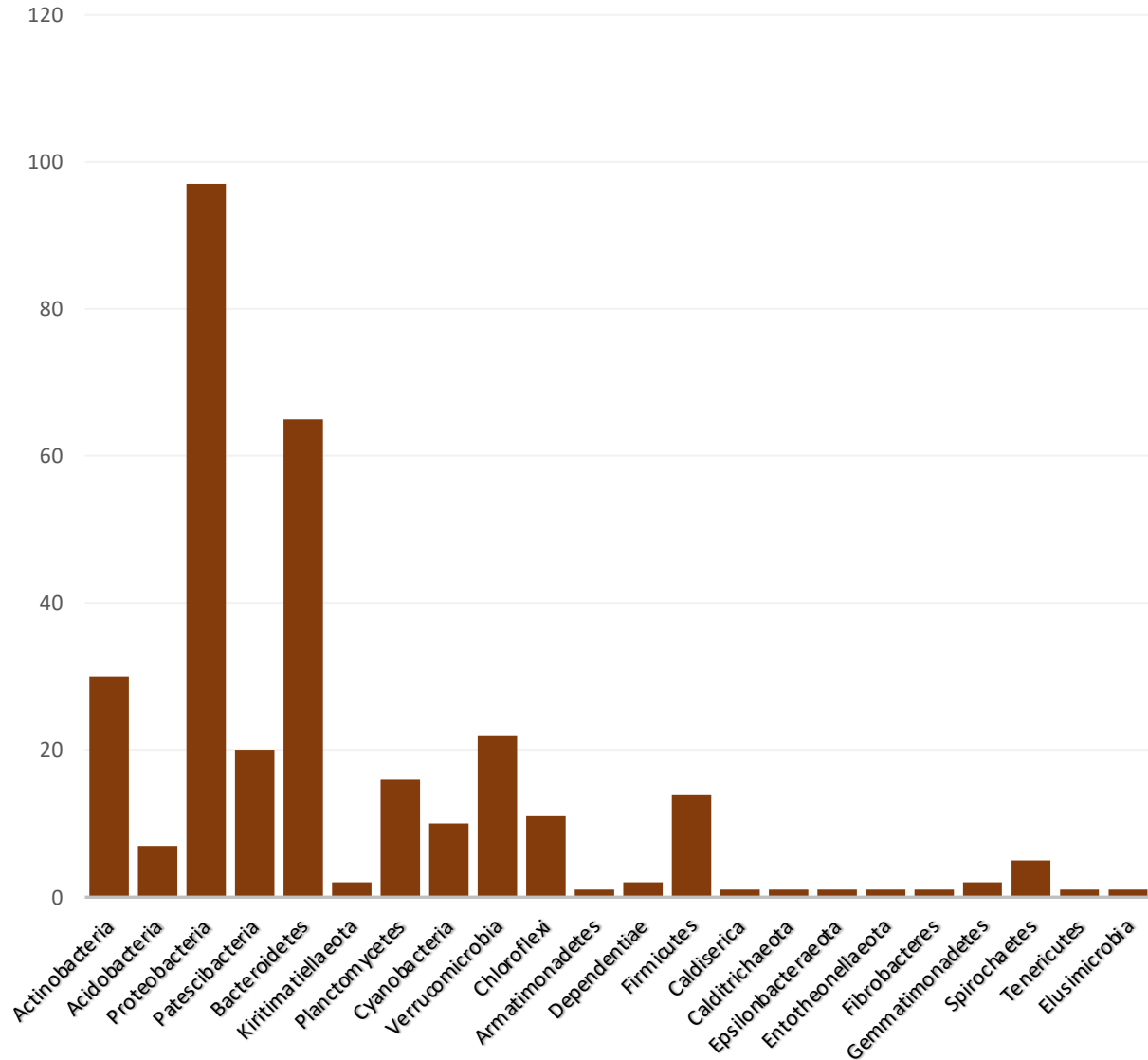

### Marine

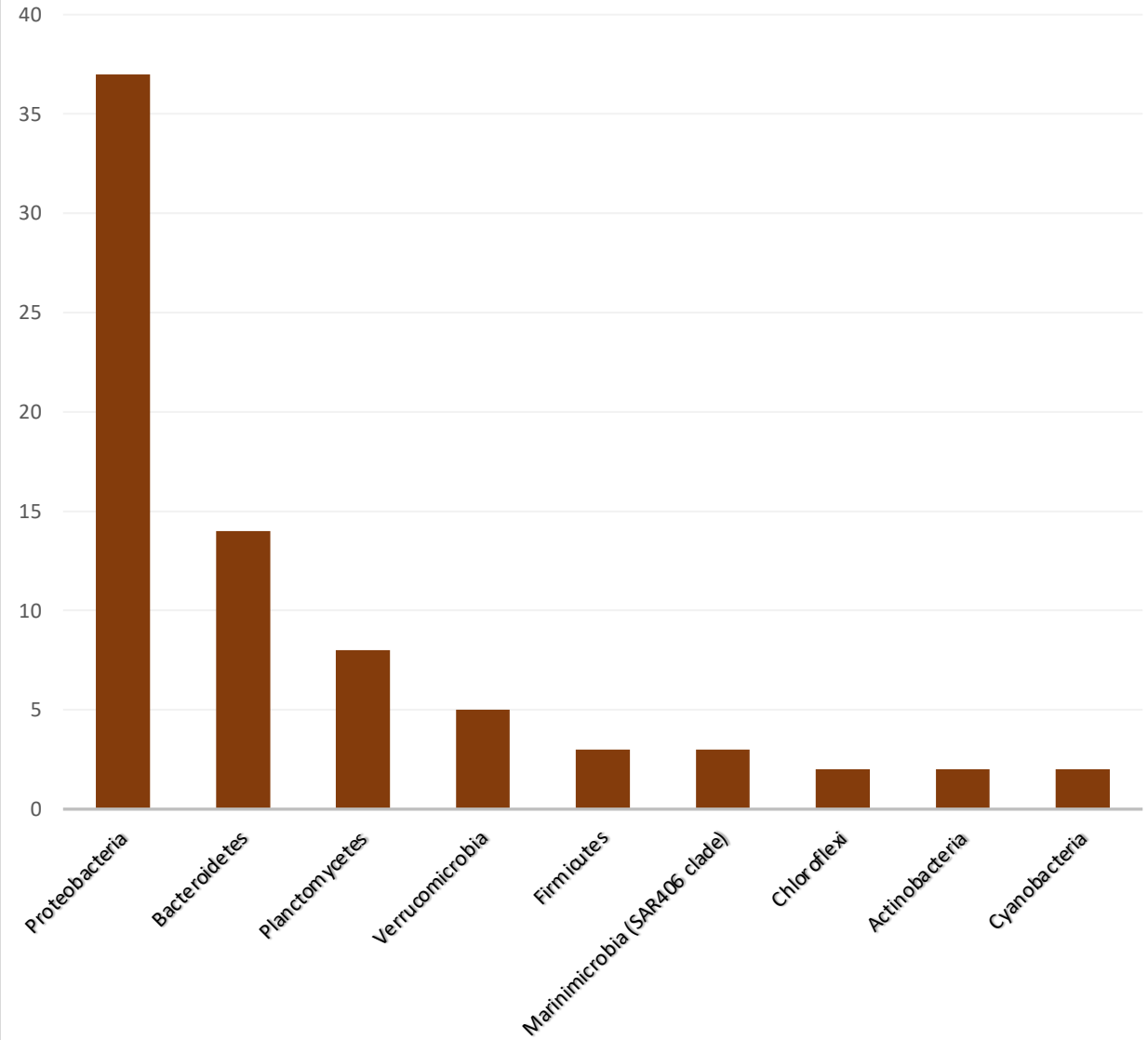

Supplement: Supplementary file 3 — Supplementary Information 3. [file 41598_2023_27816_MOESM3_ESM.pdf]

**[A]**

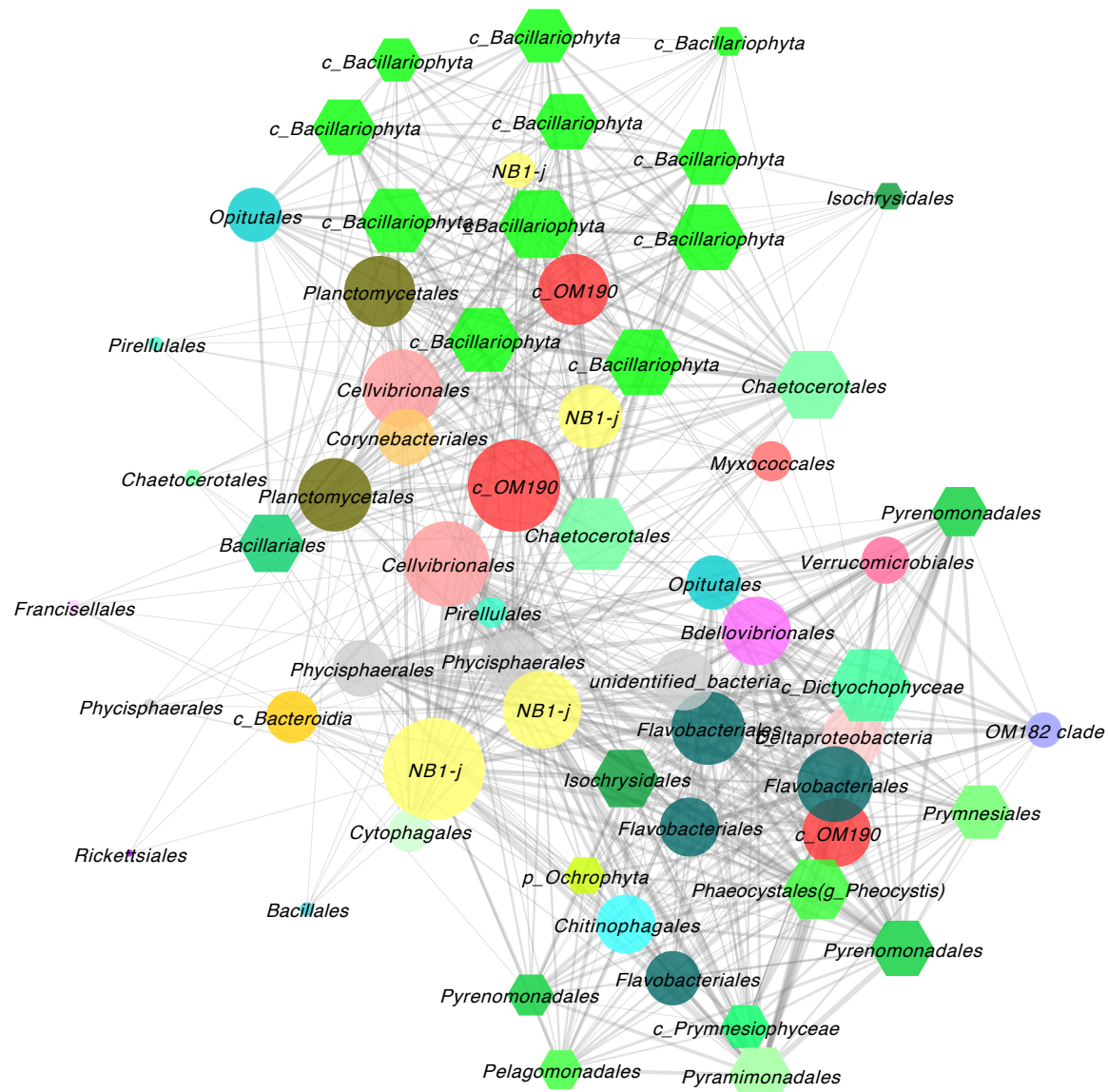

**[B]**

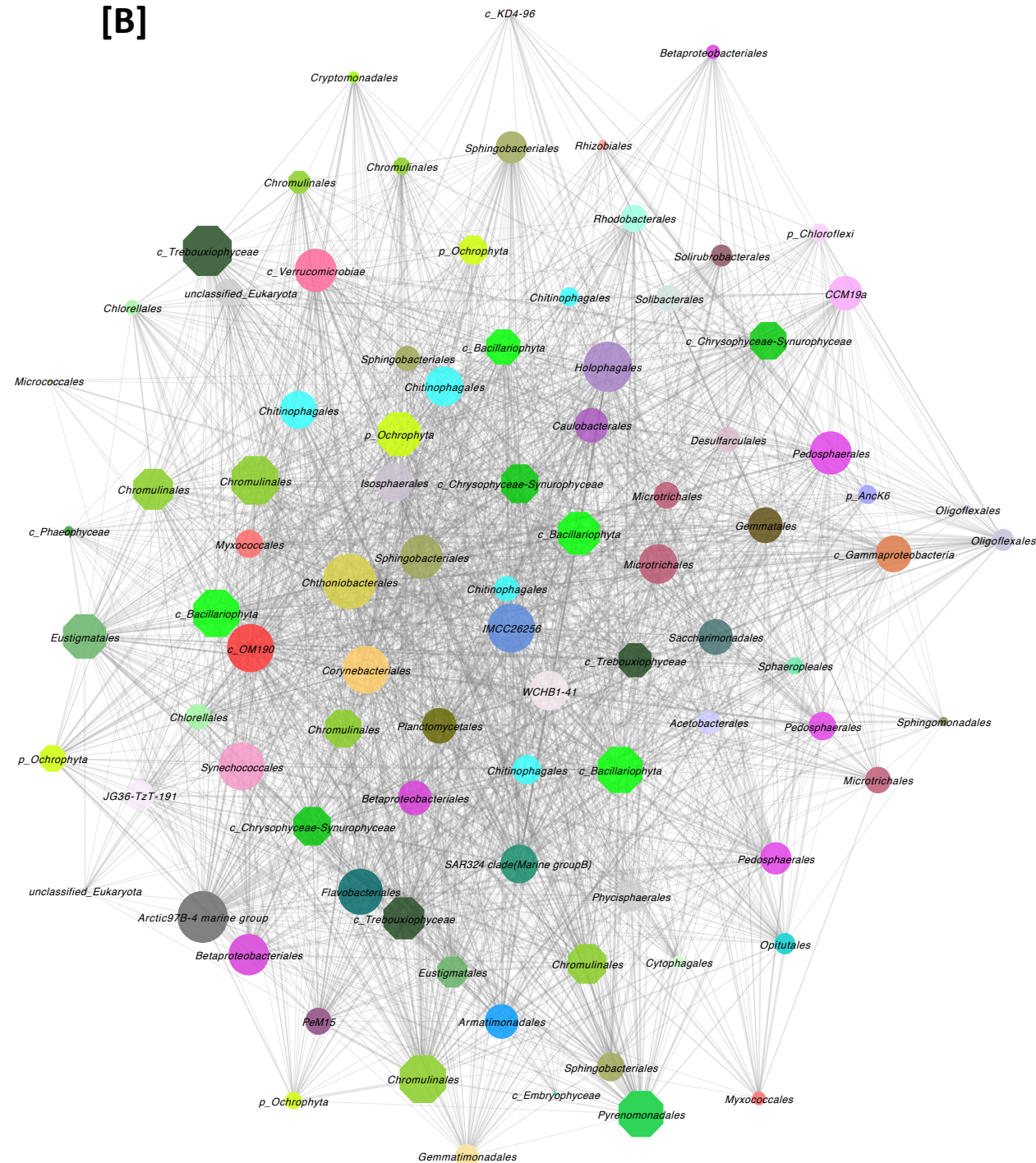

Supplement: Supplementary file 4 — Supplementary Information 4. [file 41598_2023_27816_MOESM4_ESM.pdf]
